# Preliminary results of laparoscopic cholecystectomy using real-time indocyanine green fluorescence: a cross-sectional study

**DOI:** 10.1097/MS9.0000000000000261

**Published:** 2023-02-17

**Authors:** Van Quang Vu, Van Thanh Le, Hoang Ngoc Anh Nguyen, Kim Khue Dang, Mong Vu Anh Luong

**Affiliations:** a Department of Hepato-Biliary-Pancreatic Surgery and Liver Transplant, The 108 Military Central Hospital, Hanoi, Vietnam; b College of Health Sciences, VinUniversity, Hanoi, Vietnam

**Keywords:** cholecystectomy, ICG, indocyanine green

## Abstract

**Objective:**

Evaluating the results of laparoscopic cholecystectomy (LC) using indocyanine green (ICG) fluorescence.

**Materials and methods:**

This is a cross-sectional study of patients with LC using real-time fluorescent ICG to treat gallbladder disease from May 2021 to May 2022 in the 108 Military Central Hospital.

**Results:**

There were 68 patients who underwent LC using intraoperative ICG fluorescence for bile duct visualization. The mean age of the patients was 55.4±16.2, and the male/female ratio was 1.52. Chronic cholecystitis caused by stones accounted for the majority (51.47%). The authors detected 7.35% of cases with anatomical changes of the extrahepatic biliary tract using ICG fluorescence and clearly identified the anatomy of the common bile duct and the cystic duct at 100 and 92.65%, respectively. The average surgical time was 42.8±14.6 min. There were no postoperative complications or side effects from ICG; the average hospital stay was 2.8±1.5 days.

**Conclusions:**

ICG fluorescence cholangiography allows surgeons to easily identify critical anatomical landmarks in the LC. Thereby helping the surgery to be performed safely, avoiding severe complications due to damage to the biliary tract.

Highlights

- Many authors and associations have proposed strategies to perform laparoscopic cholecystectomy safely. We proposed a routine strategy for safer laparoscopic cholecystectomy, especially in cases with adhesion.
- Indocyanine green fluorescent can identify and critical anatomical structures: cystic duct, common bile duct, and artery.
- Real-time indocyanine green fluorescent cholangiography is safe and simple, cost-effectiveness, so can quickly apply, not interrupted the operation.

## Introduction

Laparoscopic cholecystectomy (LC), a minimally invasive surgery for removing a diseased gallbladder, has been performed since 1985 by Eric Mühe (Germany). LC is currently considered the treatment of choice for benign gallbladder diseases and is one of the most common operations. Each year, there are ~60 000 cases in Japan and ~750 000 cases in the United States^1^,^2^.

According to statistics, the common bile duct (CBD) injury rate in LC ranges from 0.1 to 1.5%^3^,^4^. However, this is a serious complication that has an impact on patients’ treatment outcomes and quality of life of patients^4^,^5^. There are a number of recommendations to lower the risk of CBD injury intraoperatively, including clear dissection to get a critical view of safety, demonstrating the anatomical landmarks of the gallbladder and surrounding organs, and a cholangiogram during surgery^2^^.^

Since the 1970s, indocyanine green (ICG) has been studied and applied in medicine. Upon its intravenous administration, ICG binds to intravascular plasma proteins and becomes fluorescent. It is not metabolized in the body and is almost completely excreted from the bile. The fluorescence imaging of the biliary tract by a handheld camera can be coupled to the laparoscopic tower^6^. Thus, ICG is an ideal substance and is practical, safe, and simple to apply to clearly identify landmarks of the gallbladder and its relations, which can reduce the complications, particularly, biliary tract injury. In 2009, ICG fluorescence cholangiography intraoperatively was originally reported by Ishizawa *et al*. (Japan)^7^. Since then, this method has been put into practice in LC at major centers all over the world^1^,^4^.

In Vietnam, gallbladder diseases are becoming increasingly common, according to data from large surgery centers. LC is the first surgical option for benign gallbladder diseases. The use of ICG fluorescence imaging in LC to recognize significant anatomical features and lower the risk of intraoperative biliary tract injury has not yet been the subject of published studies or reports. Therefore, we conducted this study with the aim of determining the effectiveness of LC using ICG at 108 Military Central Hospital.

## Materials and methods

This prospective cross-sectional study enrolled all patients who underwent LC for gallbladder disease with intraoperative ICG fluorescent cholangiography at 108 Military Central Hospital from May 2021 to May 2022. The study was approved by the Institutional Ethical Committee with number 4896/QD-BV and strictly followed the Declaration of Helsinki.

The study is fully compliant with STROCSS guidelines 2021 criteria^8^ and registered with the Research Registry with the number: researchregistry8605 (https://www.researchregistry.com/browse-the-registry#home/registrationdetails/63ae8199b0decc00124fbfc4/).

### Inclusion criteria

LC using ICG fluorescent imaging in patients:

1. Acute or chronic cholecystitis with or without stones.
2. Symptomatic or large gallstones.
3. Acute pancreatitis due to gallstones has been stabilized.
4. Multiple or large gallbladder polyps or benign gallbladder tumors.

#### Exclusion criteria

Contraindications to LC:

1. Unable to insufflate CO_2_ gas into the peritoneal cavity.
2. Contraindication to general anesthesia.
3. Severe coagulopathy.
4. Suspected gallbladder cancer.

Contraindications to ICG:

1. Hypersensitivity to ICG, sodium iodine, or iodine; or had side effects with ICG previously.
2. Hyperthyroidism, hypothyroidism, and benign thyroid tumors (the iodine component in ICG may cause unwanted interactions with the treatment)^6^.

### Operation procedure

Patient preparation includes completing the patient record, performing blood tests, performing preoperative diagnostic imaging, and assessing the patient’s overall condition. ICG (Verde 2.5 mg/1 ml) was administered intravenously at a dose of 0.1 mg/kg body weight 10 h before elective cases and 1 h before emergency cases.

Equipment preparation includes the Karl Storz laparoscopic system with integrated near-infrared (NIR) light source mode and ICG fluorescent image acquisition, as well as standard laparoscopic and suturing equipment.

1. Step 1: abdominal access, evaluation of the gallbladder, and extrahepatic biliary tract.

- Trocars placement: 10 mm at the umbilicus, 10 mm in the epigastrium, and 5 mm in the right lower quadrant. CO_2_ gas insufflation maintains intra-abdominal pressure between 10 and 12 mm Hg. The patient was placed in a reverse Trendelenburg position and inclined to the left.
- Evaluating the gallbladder and extrahepatic biliary tract.
  1. Step 2: ICG fluorescence cholangiography to locate important anatomical landmarks.
    Expose the gallbladder and hepatic pedicle and switch to NIR/ICG mode to observe the extrahepatic biliary tract under ICG fluorescence. The dissection path can then be oriented while also identifying key anatomical landmarks to check for anatomical variations, biliary tract and cystic duct dilatation, inflammation, and gallstones lodged in the gallbladder neck.
  2. Step 3: Mobilize Hartman’s Pouch and dissect Calot’s triangle
    Expose Calot’s triangle. If it is difficult to observe, administer intravenously a second dose of Verdye (ICG) with a dose of 1–2 ml of slow rate, observe under NIR/ICG mode, and identify the gallbladder artery after injection in 10–30 s.
    Be aware that the total daily dose of ICG should not exceed 5 mg/kg body weight.
  3. Step 4: cut the gallbladder
    Hemoclip ligation of the cystic artery and cystic duct.
    Gallbladder removal from the liver bed. If anatomical variants are suspected during this step, we switch to NIR/ICG mode to see if an accessory biliary tract or a posterior segmental hepatic duct is draining into the gallbladder.
    Placing the gallbladder in the laparoscopic retrieval bag, cleansing the abdominal cavity, and carefully controlling the bleeding.
  4. Step 5: re-check and close the incision

Switching to NIR/ICG mode to check for bile leakage; if necessary, reinforce with sutures or hemoclips.

Removing the gallbladder via the umbilical trocar, releasing CO_2_ gas, and then closing the incision.

#### Study outcomes

The primary outcome is intraoperative and postoperative complications. We recorded the age, sex, diagnosis, and identification of anatomical landmarks under ICG fluorescence imaging, extrahepatic biliary tract anatomical variations, surgery difficulties, operation duration, and intraoperative ICG injection to locate the cystic artery.

#### Data analysis

Data was analyzed by using SPSS 26.0 software. Summary tables containing relevant variables were produced and presented as frequency, mean, and percentage.

## Results

From May 2021 to May 2022, there were 68 patients who had LC using ICG fluorescent imaging at 108 Military Central Hospital. The patients’ demographics and diagnoses are summarized in Table 1. Most of the patients were men (60,29%) with mean age of 55,4. Most of the patients presented with chronic cholecystitis due to gallstones (51.47%) (Tables 2 and 3).

**Table 1:** Patient demographic and indications for cholecystectomy (*n*=68)

Features	Results [*n* (%)]
Mean age	55,4±16,2 (22 – 84)
Sex	
Male	41 (60.29)
Female	27 (39.71)
Diagnosis	
Chronic cholecystitis due to gallstones	35 (51.47)
Acute cholecystitis due to gallstones	28 (41.18)
Having cholecystostomy	11 (16.18)
Gallbladder polyps	3 (4.41)
Gallbladder adenomyomatosis	2 (2,.94)

**Table 2:** Intraoperative characteristics (*n*=68)

Features	Results [*n* (%)]
Anatomical landmarks identification under ICG fluorescent imaging	
Common bile duct	68 (100)
Cystic duct	63 (92.65)
Cystic artery	62 (91.18)
Anatomical variants identification under ICG fluorescent imaging	5 (7.35)
Accessory bile duct	1 (1.47)
Low confluence of the cystic duct	2 (2.94)
Double gallbladder	1 (1.47)
Gallbladder bed on the left side	1 (1.47)
Intraoperative ICG administration to identify cystic artery	15 (22.06)
Difficulty to expose gallbladder	8 (1168)
Operation duration (min)	42.8±14.6 (20–80)

ICG, indocyanine green.

**Table 3:** Operation duration and its related factors

Related factors	Operation length (min)
Acute inflammation	52.63±10.59
No acute inflammation	35.0±12.51
Biliary variants	61.67±16.07
No biliary variants	41.38±13.63
Intraoperative ICG administration to identify cystic artery	61.67±11.99
No administration	37.79±10.60

ICG, indocyanine green.

The average operation time is 42.8 min. In all cases, we can easily see CBDs by ICG fluorescent. The cystic duct and artery could not be visualized in five and six cases, respectively. We found five anatomic variants of the extrahepatic biliary system: one with an accessory bile duct, two with low confluence of the cystic duct, one with a double gallbladder, and one with a gallbladder bed on the left side. We encountered eight cases with difficulty exposing the gallbladder due to severe adhesion.

In our study, 12 patients (17.65%) required subhepatic drainage. No patient had a conversion to open surgery. There was no intraoperative biliary tract or vascular injury, no patient experienced postoperative complications, and no patient experienced ICG side effects. The average hospital stay was 2.8±1.5 days (2–10 days).

## Discussions

In hepatobiliary surgery, identifying anatomical features, particularly arteries and the extrahepatic biliary tract variations, is essential because, if done improperly, the patient could experience serious complications. Numerous techniques have been developed to detect the intraoperative biliary tract, including cholangiography and endoscopic ultrasound. These techniques do have certain disadvantages, such as the necessity of bulky, expensive equipment, the need for additional personnel during the operation, the lengthening of the operation, and the exposure of patients and medical personnel to radiographs^2^,^9^.

ICG is an inactive, nonradioactive, user-safe, and cost-effective tracer. ICG strongly binds to plasma when administered intravenously and fluoresces. It is subsequently stored in the liver parenchyma and excreted in the bile without being metabolized. Since the 1970s, ICG has been used in medicine, with particular uses in ophthalmology, lymph node dissection for breast cancer, gastrointestinal cancer, cholangiography, hepatectomy, liver transplantation, and other fields. Recent years have seen a significant increase in the use of ICG in hepatobiliary surgery to detect the biliary tract, blood vessels, and lymphatic system, as well as assess liver function^6^.

According to our study of 68 patients who underwent LC using fluorescein ICG, the patient’s average age was 55.4±16.2 years, which is similar to the findings of the authors Shibata *et al*.^5^ is 62.8 years old, and by Hiwatashi *et al*.^1^ is 61.34 years old on average. Males were the majority of participants (60.29%), with a male/female ratio of 1.52, comparable to Shibata’s findings^5^ of 1.4. All of the gallbladder diseases in our study were benign, with chronic gallstone cholecystitis making up the majority (51.47%), followed by acute gallstone cholecystitis (41.18%) – no venous drainage (25.0%) and drained gallbladder (16.18%). Author Hiwatashi *et al*.^1^ reported similar results: 89.2% of patients had gallstones, and 49.2% of patients had acute cholecystitis. Author Ambe *et al*.^10^ report that the rate of acute cholecystitis in the group receiving ICG is 13.8%.

**Figure 1:**
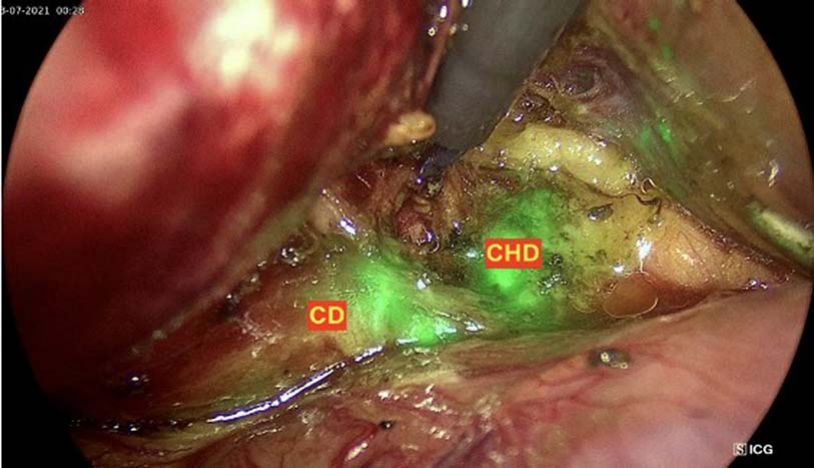
Biliary tree anatomical landmarks under indocyanine green fluorescence in patients with chronic cholecystitis. CD, cystic duct; CHD, common hepatic duct.

**Figure 2:**
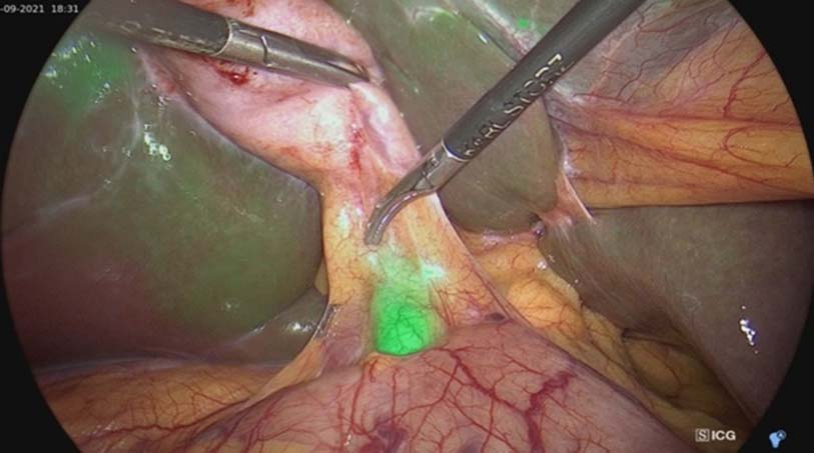
In a case of acute cholecystitis, indocyanine green fluorescein does not enter the gallbladder; only the cystic duct and common bile duct are visible.

In a LC, ICG fluorescence is an emerging technique to help the surgeon see critical anatomical landmarks such as the cystic artery, cystic duct, common hepatic duct, CBD, and bile duct. Understanding the extrahepatic biliary tract’s anomalies can assist in minimizing the damage done to it. ICG is known to be excreted in bile; hence, the extrahepatic biliary tract can be seen by ICG fluorescence imaging. However, the limitation of this approach is that ICG fluorescence cannot be seen when the tissue is thicker than 1 cm^6^. It can be challenging to see the ICG fluorescence in acute cholecystitis caused by stones because of edema and severe inflammation of the tissues surrounding the gallbladder, the cystic duct, and the hepatic pedicle. In our study, we could clearly see the CBD in 100% of patients, the cystic duct in 92.65%, and the cystic artery in 91.18% using ICG fluorescence imaging. In patients whose cystic duct and cystic artery could not be seen under ICG fluorescence, there were all cases of acute cholecystitis with or without gallbladder drainage. According to our study, only 11.68% of patients with acute cholecystitis had difficulty dissecting the cystic duct; nevertheless, after dissection, ICG fluorescence was clearly evident. In Ishizawa *et al*.’s study^7^, 9 out of 10 cases of LC using ICG fluorescence detected the cystic duct. Compared to Shibata *et al*.’s^5^ study of 12 patients who had preoperative intravenous ICG administration, 83.3% of patients had observed cysts and CBDs. Hiwatashi *et al*.^1^ reported that 83.1 and 93.8%, respectively, of patients could observe the cystic duct and CBD using ICG fluorescence. Shibata *et al*.’s study^5^ recommended that biliary ICG fluorescence could be observed in patients with a BMI of 30.4 kg/m^2^. Additionally, in the study, we administered the second dose of ICG intraoperatively through a peripheral vein to identify the cystic artery in 22.06%, avoiding mistaken clamping with the cystic duct or artery omission (Figs 1 and 2).

A further benefit of ICG is the ability to detect biliary tract anatomical alterations during LC. In the study, five cases (7.35%) with abnormal biliary tract variations were identified. While dissection revealed Calot’s triangle, we found two patients (2.94%) with a low cystic duct based on ICG fluorescence. Both cases were dissected along the cystic duct using ICG fluorescence as a guide to avoid damage to the CBD and prevent leaving the cystic duct stump for too long. When separating the gallbladder from the gallbladder bed, we found one case (1.47%) with a segment of tissue that was abnormally thickened and continuous with the gallbladder. Through ICG fluorescence, we discovered it to be an extra bile duct that entered the gallbladder from the liver parenchyma; we cut it out and closed it with PDS 5.0 sutures to prevent bile leakage. In another case (1.47%), the left gallbladder bed was discovered after entering the abdomen adjacent to the round ligament. In this case, we prevented injury to the main bile duct and hepatic artery by using fluorescent ICG to observe the course of the cystic duct. Since the cystic duct traveled behind the common hepatic duct and in front of the hepatic artery, we decided to cut it close to the left border of the common hepatic duct. Additionally, there was one case (1.47%) of the double-chambered gallbladder, which was comparable to author Pesce *et al*.’s^11^ 8% and less than Ishizawa *et al*.’s^3^ 8/52 cases (15.4%).

The average length of operation was 42.8±14.6 min, which was similar to the findings of earlier studies^3^–^5^. The endoscopic camera (made by the Karl Storz firm) has recently been coupled with the capability to transmit NIR light sources, making surgery more convenient. Because of the handheld equipment’s compact size, lightweight, cost-effectiveness, and ease of switching between imaging modalities, the length of the operation is not significantly longer compared to the group not receiving ICG cholangiography. According to the study by Ambe *et al*.^10^, there was no difference in surgical time between the groups who utilized fluorescein ICG cholangiography and those who did not (53 vs. 54 min).

## Conclusions

LC using ICG fluorescence provided positive findings when there were no cases of hepatic artery and common biliary tract injury, identified 7.35% of patients with extrahepatic biliary tract abnormalities, identified the CBD and cystic duct anatomy in 100 and 92.65% of cases, respectively, no postoperative complications, and no ICG side effects were noted.

In cases of acute cholecystitis, it is important to dissect the surrounding fatty tissues to observe the ICG fluorescence and locate anatomical landmarks clearly. However, when the inflammatory tissue is thicker than 1 cm, it is impossible to observe under ICG fluorescence.

## Ethical approval

The study was approved by the Institutional Ethical Committee with number 4896/QD-BV.

## Consent

None.

## Sources of funding

The study was not received external fund.

## Author contribution

L.V.T.: study design and oversight all the study. V.V.Q.: data analysis and interpretation. N.H.N.A.: data collection and write the paper. L.M.V.A.: prepare and write the paper. D.K.K.: edit, revise, and prepare for submitting the paper.

## Conflicts of interest

All authors have no conflicts of interest to disclose.

## Research registration unique identifying number (UIN)

1. Name of the registry: Research Registry.
2. Unique identifying number or registration ID: researchregistry8605.
3. Hyperlink to your specific registration (must be publicly accessible and will be checked): https://www.researchregistry.com/browse-the-registry#home/registrationdetails/63ae8199b0decc00124fbfc4/.

## Guarantor

Le Van Thanh.

Vu Van Quang.

## Provenance and peer review

Not commissioned, externally peer-reviewed.
